# miRNAs in acute myeloid leukemia

**DOI:** 10.18632/oncotarget.12343

**Published:** 2016-09-29

**Authors:** Qiong Liao, Bingping Wang, Xia Li, Guosheng Jiang

**Affiliations:** ^1^ Key Laboratory for Rare & Uncommon Diseases of Shandong Province, Institute of Basic Medicine, Shandong Academy of Medical Sciences, Jinan, Shandong, P.R. China; ^2^ School of Medicine and Life Sciences, Jinan University, Jinan, Shandong, P.R. China; ^3^ Department of Hematology, Shengli Oilfield Central Hospital, Dongying, Shandong, P.R. China; ^4^ Shandong University School of Medicine, Jinan, Shandong, P.R. China

**Keywords:** miRNAs regulatory signaling pathways, acute myeloid leukemia, pathogenesis, prognosis, miRNA-directed therapy

## Abstract

MicroRNAs (miRNAs) are small, non-coding RNAs found throughout the eukaryotes that control the expression of a number of genes involved in commitment and differentiation of hematopoietic stem cells and tumorigenesis. Widespread dysregulation of miRNAs have been found in hematological malignancies, including human acute myeloid leukemia (AML). A comprehensive understanding of the role of miRNAs within the complex regulatory networks that are disrupted in malignant AML cells is a prerequisite for the development of therapeutic strategies employing miRNA modulators. Herein, we review the roles of emerging miRNAs and the miRNAs regulatory networks in AML pathogenesis, prognosis, and miRNA-directed therapies.

miRNAs are small, non-coding RNAs that bind target mRNAs leading to their degradation or disruption of their translation [1]. Because regulation of specific gene transcripts by miRNAs is essential to many developmental processes, it is not surprising that dysfunctional miRNA signaling contributes to diseases that arise from defective cellular proliferation and differentiation including acute myeloid leukemia (AML). AML is a highly heterogeneous disease characterized by failure of myeloid precursor cells to undergo terminal differentiation from proliferating precursor cells into mature blood cells. In addition to genetic and genomic aberrations, including chromosome translocations and inversions, gene deletions and mutations, changes in post-transcriptional regulation by miRNAs in AML have been reported. These miRNAs have functional relevance and some are sufficient to cause a cascade of downstream effects in malignant AML transformation in animal models, indicating the potential of miRNA-based therapeutic strategies. However, aberrant miRNAs are extensively associated with genetic and genomic alterations seen in AML. A comprehensive understanding of the full network of oncogenic events and downstream signaling pathways related to altered miRNAs could reveal new therapeutic strategies to modulate these miRNAs in AML.

## THE ROLE OF MIRNAS IN PATHOGENESIS OF AML

Recent studies have linked disruptions of miRNAs involved in gene regulation to AML pathogenesis. For example, *miR-155* is upregulated in bone marrow of AML patients with mutations in nucleophosmin *(NPM1)* and *FLT3-ITD* [2, 3]. Overexpression of *miR-155* in human hematopoietic stem cells increased proliferation of myeloid progenitors and blocked differentiation into erythrocyes and megakaryocytes. Overexpression of *miR-155* in murine lymphocyte precursors induced polyclonal lymphocytosis but also led to high-grade lymphocytic leukemia [4, 5]. Src homology-2 domain containing inositol 5-phosphatase 1 (*SHIP1*), a negative regulator of immune cell signaling, is directly bound and inhibited by *miR-155* [4, 6]. Mice lacking *SHIP1* or overexpressing *miR-155* exhibit similar myelo-proliferative disease characteristics, including increased granulocyte-monocyte cells and reduced B-lymphocytes [7]. Interestingly, loss of function *SHIP1* mutations have been found in a small number of AML patients and have been linked to oncogenesis [8]. *miR-155* also targets *CEBPB* [4], which is critical in granulopoiesis [9], suggesting that aberrant *miR-155* signaling could deregulate both *SHIP1* and *CEBPB* in AML*.*

The *miR-125* family exists as three homologues (*miR-125a*, *b*, and *c*) all on different chromosomes. While studies of *miR-125a* suggested it has a tumor suppressor role in AML, *miR-125b* is considered as an oncomiRNA. *miR-125a* expression in cytogenetically normal AML (CN-AML) was most decreased in favorable and intermediate prognostic populations and associated with decreased survival [10]. In contrast, *miR-125b* is highly expressed in hematopoietic stem cells and when ectopically overexpressed leads to development of leukemia. The leukemic subtype developed is dependent on the level of *miR-125b*. Over-expression of *miR-125b* can block the terminal differentiation of HL60 and NB4 AML cell lines *in vitro* [11, 12]. *miR-125b* exists as two paralogs, *miR-125b1* and *miR-125b2*, which contain the same seed region but are located on different chromosomes and are therefore independently regulated. *MiR-125b-1* is strongly up-regulated in AML blasts, in particular those from patients with the t(2:11)(p21:q23) translocation, which shows a 90-fold increase [11-13]. In a study of CN-AML patients 60-year or older, *miR-125b2* was one of the most overexpressed miRNAs of the 32 differentially expressed miRNAs examined [14]. *miR-125b* targets the pro-apoptotic *Bak1* and *Bmf* transcripts and also negatively regulates *p53* and many other genes in the p53 pathway, providing one mechanism for *miR-125b*'s role as an oncomiR in AML. In addition, *NOTCH1* is also a target of *miR-125b.* While *NOTCH* activation plays oncogenic roles in acute T-lymphoblastic leukemia (T-ALL), *NOTCH* is suppressive for AML cell growth depending on cell conditions [15]. Romero et al. recently observed that C/EBPα binds directly to the promoter of *miR-125b,* increasing its transcription rate and subsequently reducing *NOTCH1* levels in AML [16].

*miR-100* is another oncomiR in AML with high expression in primary AML blasts [17]. The up-regulation of *miR-100* represses its target RB serine phosphatase (*RBSP3)* [18]*,* which is a phosphatase-like tumor suppressor, frequently mutated in human hematopoietic cell lines. RBSP3 can influence the phosphorylation status of pRB and then the release of E2F1 in controlling cell cycle. *In vitro* studies revealed that the mechanism underlying *miR-100* in arresting human granulocyte and monocyte differentiation and promoting cell survival was through the RBSP3-pRB-E2F1 pathway [18].

Conversely, several miRNAs have been characterized as tumor suppressors in AML. Wang X, et al [19] saw that *miR-29a* and *miR-142-3p* were significantly increased in peripheral blood mononuclear cells (PBMNCs) and bone marrow (BM) white blood cells from AML patients. Increased *miR-29a* or *miR-142-3p* leads to increased differentiation into granulocytes and monocytes, while reduction of either *miR-29a* or *miR-142-3p* suppressed myeloid differentiation in leukemia cell models. Furthermore, co-transfection of both *miR-29a* and *miR-142-3p* inhibited both their common target, cyclin T2 (*CCNT2*), and their individual target genes cyclin-dependent kinase 6 (*CDK6*) and TGF-β activated kinase 1/MAP3K7 binding protein 2 (*TAB2*), respectively, leading to a larger synergistic reduction in myeloid differentiation [19-21]. *miR-29a* and *miR-142-3p* promote monocytopoiesis by suppressing *CCNT2,* which reduces pRb protein levels and cell proliferation. CDK6 interferes with Runx1 binding to DNA and C/EBPα in immature proliferating cells blocking their myeloid differentiation. Removal of CDK6 inhibition selectively activates Runx proteins, promoting terminal cell differentiation. *miR-142-3p* inhibits *TAB2* expression and therefore an increase in *miR-142-3p* pushes monocytic precursors to differentiate into macrophages rather than osteoclasts. More recently, Gong et al. [22] reported that all the *miR-29* family members, *miR-29a*, *-29b* and *-29c*, were reduced in PBMNCs and bone marrow CD34+ cells from AML patients. Reintroducing each *miR-29* member into AML BM blasts was able to partially correct abnormal cell proliferation and apoptosis repression and myeloid differentiation arrest. *Akt2* was also identified to be a target of the three *miR-29* members that was significantly increased in the AML blasts. In myeloid leukemogenesis, overexpressed *c-Myc* inhibits *miR-29* family expression, resulting in increased Akt2 and CCND2 protein expression in AML [20, 22-24].

A most recent study that profiled differentially expressed miRNAs from AML patients’ granulocytes versus healthy subjects identified two significantly under-expressed miRNAs *miR-26a-5p* and *miR-23b-3p* [25]. These two miRNAs have a common target the *PrxIII* gene. The accumulation of PrxIII caused by decreased *miR-26a-5p* and *miR-23b-3p* led to a considerable decrease in reactive oxygen species (ROS) in primary AML granulocyte samples and transfected cells [25]. Emerging evidence suggests that the ROS signal plays a critical role in regulating the balance between self-renewal and differentiation of hematopoietic stem cells (HSCs) [26]. The decreased ROS levels might push HSCs toward differentiation into myeloid lineage fates in hematopoietic systems, providing one mechanism for *miR-26a-5p* and *miR-23b-3p’*s role as tumor suppressors.

*miR-223* was previously characterized as a negative regulator of AML pathogenesis [27]. In support of this, the *miR-223* mutant mouse was found to exhibit granulocytosis and hemizygous loss of the *miR-223* gene has been identified in AML patients [28]. Further studies identified that transcription factor myocyte-specific enhancer factor 2C (*MEF2C*) gene is one of the targets of *miR-223* in mediating its anti-proliferative effects in granulopoiesis, while *miR-223* transcription is activated by *RUNX1/RUNX1T1-*induced chromatin remodeling. Recently, Maria et al, using the miR-223^-/Y^ and miR-223^−/−^ mice, found that loss of *miR-223* alone results in a modest expansion of myeloid progenitors, but does not induce myeloproliferative disorder or alter HSC long-term repopulating and self-renewal capacity [29]. These results suggest that *miR-223* loss is not sufficient to cause AML but it may be a contributing factor in leukemogenesis that requires further study.

In addition to these miRNAs for which comprehensive studies have identified roles in AML pathogenesis and mechanism of actions, several new AML miRNAs have been highlighted in recent publications. For example, *miR-9* (*miR-9-5p*) and miR-9* (*miR-9-3p*) are highly conserved miRNAs produced from a single precursor. Overexpression of *miR-9* has been shown to enhance transformation of murine hematopoietic progenitor cells by MLL-AF9 [30]. Also, *miR-9* targeting of the *LIN28B/Let-7/HMGA2* axis induces monocytic differentiation in KASUMI-1 cells [31]. High expressions of both *miR-9* and 9* (miR-9/9*) were detected in most cases from a cohort of 647 primary AML patients. Their expression levels varied among different AML subtypes with the highest expression in MLL-related leukemias harboring 11q23 abnormalities and in normal karyotype AML cases with mutations in *NPM1* [32]. Ectopic expression of *miR-9* or miR-9* blocked neutrophil development in the myeloid 32D cell line and in mouse primary lineage-negative bone marrow cells by inhibiting ETS-related gene (*ERG*) [32].

Studying AML caused by toxic DNA interstrand crosslinks (ICLs), Alemdehy et al [33] found that *miR-139-3p* and *miR-199a-3p* had opposite effects on hematopoiesis. In the study, both *miR-139-3p* and *miR-199a-3p* increased with age in myeloid progenitors from the nucleotide excision repair gene (Ercc1)-deficient mice. Ectopic expression of *miR-139-3p* inhibited myeloid progenitor proliferation of myeloid progenitors, increased *miR-199a-3p* enhanced proliferation of progenitors and accelerated the AML phenotype. Although this study directly supports the role of *miR-199a-3p* as an onco-miRNA, it also indicates that an auto-regulatory negative feedback from the elevated *miR-139-3p* as a suppressor is involved in the defective hematopoietic function in ICL-caused AML.

*miR-638* is frequently down-regulated in various solid tumors, and it represses BaP-induced carcinogenesis by targeting breast cancer 1 (*BRCA1*) [34]. Lin et al recently saw that *miR-638* was reduced in primary AML samples vs cells undergoing normal hematopoiesis [35]. Overexpression of *miR-638* inhibited proliferation and promoted differentiation, while inhibition of *miR-638* promoted proliferation and reduced differentiation. *CDK2* has been identified as an miR-638 target and *CDK2* overexpression rescued the *miR-638-* repressed colony formation of HL-60 cells. Considering that *CDK2* is also commonly down-regulated during granulopoiesis, the *miR-638/CDK2* axis may serve as a marker for prognosis or treatment response. However, *miR-638* overexpression was not sufficient to overcome the failure of leukemic cells to differentiate. Rather, it reduced the threshold for leukemic cells to undergo forced myeloid differentiation. Thus, further studies in exploring the *miR-638* regulatory network is necessary for fully clarifying the contribution of *miR-638* to myeloid leukemia [35].

Accumulating evidence indicates that the *miR-181* family plays important roles in AML pathogenesis [36]. Expression of all *miR-181* family members was reduced in adult AML patients (M1-M3 subtypes), suggesting all function as tumor suppressors. *miR-181a* promotes a proliferative state by inhibiting terminal differentiation of both cultured HL-60 cells and CD34^+^ hematopoietic stem/progenitor cells (HSPCs). The *in vivo* expression of *miR-181* partially reversed the lack of myeloid differentiation in AML patients and in the mice with CD34^+^ HSPCs from AML patients [37]

## MIRNAS WERE ASSOCIATED WITH AML STEM CELLS

It has been demonstrated that, in normal hematopoiesis, some miRNAs were involved in progenitor lineage commitment[38] and controlling HSC [39-41] by coordinate repression of multiple targets [42]. For example, HSC self-renewal can be governed by *miR-125a/b*, *miR-29a*, and *miR-126* [40, 43-45]. Recent studies have revealed important roles for miRNAs in leukemic stem cells (LSCs), linked to long-term self-renewal and transient cell-cycle quiescence or even dormancy [46]. LSCs are linked to treatment failure, recurrence, and chemotherapy resistant disease in patients with AML [47-49]. On the other hand, LSC frequency at the time of diagnosis and after treatment are prognostic indicators of poor AML outcomes [48, 50, 51]. Elucidation of the pattern of miRNA expression could help to elucidate the molecular mechanism of early hematopoietic differentiation and the pathogenic mechanisms underlying AML [52], risk categories, and prognosis [2, 53, 54].

Several LSC-associated miRNAs are linked to the development of leukemia. For example, *miR-17-92* polycistron maintained LSC in Mixed-lineage leukemia (MLL) models [55]. Inhibiting *miR-196* or *miR-21* reduced LSCs in an experimental model of human MLL [56], *miR-126* reduction reduced AML growth, and overexpression of some miRNAs induced murine leukemic transformation [44, 57]. Recent evidence indicates that LSCs-released microvesicles (LMVs) can regulate the malignance of AML cells, overexpression of tumor suppressive *miR34a* is able to interrupt this process, which indicates that modulating *miR34a* could offer a new approach for the management of AML [58]. In the other reports, overexpression of *miR-29a* in normal hematopoietic cells caused a myeloproliferative disorder that progressed to AML [23], and overexpression of *miR-125b* led to leukemia [11].

**Figure 1:**
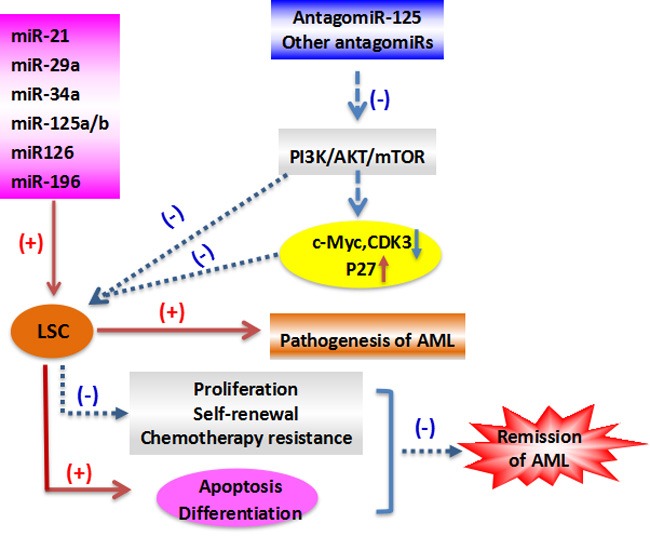
The role of LSC in the pathogenesis and remission of AML

Furthermore, *miR-126* was found to restrain cell cycle progression, prevent differentiation, and increase self-renewal of primary LSC *in vivo*, and the results demonstrate that miRNAs preserve LSC quiescence and promote chemotherapy resistance [59]. One study reported that targeting *miR-126* in leukemic cells could reduce cell growth by inducing apoptosis [60]. Lechman and colleagues reported that miRNAs were involved in human LSC function [59], and another recent study revealed that the higher levels of *miR-126* in older AML patients correlated with poor overall survival [61]. Thus, miRNA expression, including *miR-126* in LSCs, impacts on the clinical outcome of AML patients. Furthermore, overexpression of *miR-126* increased primitive quiescence in AML cells and reduced their differentiation into AML blasts. Knockdown of *miR-126* promoted led primitive AML cells to exit their quiescent stem-like state into a more committed population of progenitors with decreased capacity for self-renewal. As to the mechanism of its cell cycle regulation, *miR-126* controls the PI3K-AKT-mTOR pathway [40], a gatekeeper of LSC G0-G1 cycle control [62]. AML cells with increased m*iR-126* are more resistant to standard chemotherapy resulting in their enrichment during treatment. *MiR-126* knockdown leads to increased proliferation of HSCs but impaired maintenance of LSCs, while its overexpression promotes LSC self-renewal and inhibits expansion of HSCs [40, 59]. Many studies have profiled miRNA expression in primary AML cells [2, 3, 53, 63, 64]. Reduced *miR-126* induced AML cell apoptosis, but enhanced expansion of HSCs in normal BM. The role of *miR-126* in LSC function appears to depend on age and AML subtype [61]. miRNAs appear to provide excellent LSC therapeutic targets due to their role in transformation of normal myeloid progenitors/stem cells into AML LSCs, but targeting LSCs without harming normal HSCs remains a significant hurdle [65]. Because the above-mentioned studies indicate that high *miR-126* expression correlates with ‘stemness’ and that *miR-126* is a viable target for eliminating the LSC in AML, the therapeutic feasibility of targeting *miR-126* in LSCs is a subject of interest. Targeting the single *miR-126* in LSCs reduced their colony formation and eliminated all leukemic cells, which suggests *miR-126* as a target to specifically eradicate LSCs [60, 61]. *miR-126* inhibition reduces LSC activity and chemotherapy resistance, which when used in conjunction with conventional leukemic therapies may significantly improve patient outcomes [66]. These observations suggest that selectively targeting LSCs by inhibiting *miR-126*, while simultaneously promoting the recovery of normal HSCs is a potential new therapy for AML (Figure 1).

There are many more miRNAs have been shown in publication that may be involved in the pathogenesis of AML (Table 1), suggesting incredible enthusiasm for research in this area in recent years. However, these studies share few similarities and there are several technical considerations to help understand the results. For example, applying multiple methods in parallel to detect a miRNA or the regulation of a miRNA is more appropriate based on the existence of related miRNAs with a high degree in sequence homology. Oftentimes, the regulatory changes in miRNA levels are small and might get lost in the biological noise when using a small number of samples. Using *in vitro* systems to study miRNA phenotypes might be different from what is happening *in vivo*. Lastly, efficacy of over-expression or anti-miR tools should be validated using downstream target readout to show the endogenous interaction between the miRNA and the targets.

**Table 1:** miRNAs that are involved in the pathogenesis of AML

Subtype	Genetic subtype	Up-regulated miRNA	Down-regulated miRNA	References
AML	(15; 17)	miR-127, miR-134, miR-299-5p, miR-323, miR-376a, miR-382, miR-29b-3p, miR-224, miR-368, miR-181a-5p	miR-17-5p, miR-20a, miR-126, miR-126*, miRNA-181a-3p, miR-126-5p	[3, 6, 22, 36, 74, 96, 101-104]
AML	t (8; 21)	miR-27a, miR-126, miR-150, miR -223, miR-29b-3p	miR-126-5p	[22, 102, 104, 105]
AML	t(11q23)/MLL	miR-326, miR-219, miR-194, miR-99b, miR-328, miR-196b, miR-29b-3p	miR-34b, miR-15a, miR-29a, miR-29c, miR-372, miR-30a, miR-29b, miR-30e, miR-196a, let-7f, miR-102, miR-33, miR-299, miR-193, miR-126-5p	[2, 22, 98, 102, 104, 106-108]
CBF-AML	t (8; 21) or inv (16)	miR-126, miR-126*miR-29b-3p	miR-126-5p	[22, 53, 102, 104]
APL	14q32	miR-136-5p, miR-376a-3p, miR-29b-3p	miR-126-5p	[3, 22, 102, 104]
AML	t (9; 11) (p22; q23)	-	miR-126-5p	[102]
AML	t(6;11)(q27;q23)	miR-21, miR-26a, miR-26b, miR-29b-3p	miR-126-5p	[22, 102, 104, 109, 110]
AML	three body (+8)	miR-24a, miR-30d, miR-29b-3p	miR-126-5p	[22, 102, 104, 111]
AML	CBFB-MYH11	miR-377-3p, miR-495-3p, miR-29b-3p	miR-126-5p	[22, 102, 104]
AML	RUNX1/RUNX1T1	miR-4516, miR-4739	-	[104]
CN-AML	NPM1/HOX(+)	miR-10a, miR-10b, miR-196a-1	-	[112]
AML (no APL)	14q32	-	miR-136-5p, miR-654-3p, miR-381-3p, miR -376a-3p, miR-377-3p, miR-376c-3p, miR-495-3p	[104]

## MIRNA REGULATORY SIGNALING PATHWAYS IN AML

The associations of miRNAs with critical mutations and abnormal expressions of individual genes in AML have been nicely reviewed before [27]. It's well recognized that miRNAs do not function through a single gene target and the combined regulation of many different genes determines the functionality of a miRNA. Recent progress sheds more lights on the systemic level miRNAs regulatory signaling networks in AML.

**Figure 2:**
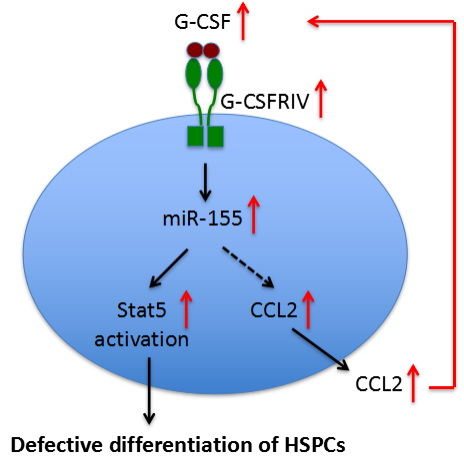
A positive-feedback mechanism involving *miR-155* in defective differentiation of HSPCs in AML

Pediatric AML patients with overexpressed granulocyte stimulating factor receptor class IV (G-CSFRIV), a major regulator of granulopoiesis [67], have been associated with defective differentiation and relapse risk. Zhang et al [68] identified that elevated *miR-155* expression and reduced levels of *miR-155* target genes (*PU.1*, *GFI-1* and *TP53INP1*) were associated with a sustained Stat5 activation in G-CSF-stimulated HSPCs isolated from AML patients with G-CSFRIV overexpression. In addition, these HSPCs secreted more chemokine (C-C motif) ligand 2 (CCL2), a strong chemotactic factor for monocytes and macrophages, and the CCL2 levels were correlated with Stat5 activation and high *miR-155* expression. *miR-155* was found to indirectly regulate *CCL2* expression [69] and CCL2 deficiency was shown to impair the secretion of G-CSF [70]. These observations clearly indicate the leukemogenic role of the G-CSF/G-CSFRIV−Stat5−miR-155−CCL2−G-CSF positive feedback loop in AML, and also pointed out the necessity in developing personalized effective anti-leukemia treatments for patients with increased G-CSFRIV, who are more susceptible to G-CSF-induced upregulation of *miR-155* and subsequent *de novo* leukemogenicity or relapse (Figure 2).

**Figure 3:**
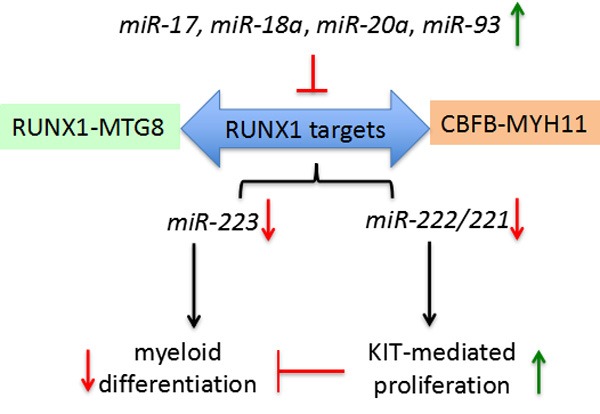
miRNAs (*miR-17, miR-18a, miR-20a, miR-93*) negatively regulate RUNX1 targets, including *miR-223* and *miR-222/221*, in blocking myeloid differentiation by increasing KIT expression and enabling KIT-mediated proliferation

The two most common AML chromosome rearrangements generate the RUNX1-MTG8 (AML1-ETO) and CBFB-MYH11 fusion proteins. Both fusions are dominant negative on RUNX1 function. *miR-223,* one RUNX1 target, is critical for the establishment of granulocyte and monocyte lineages [71, 72]. The *miR-222/221* gene cluster*,* also a RUNX1 target, regulates the kitproto-oncogene protein (*KIT)* receptor by targeting its 3′-UTR [73]. In (CBF)-AML samples with reduced *miR-221* and *miR-222* there is a concomitant up-regulation of KIT and KIT-induced proliferation [74]. KIT up-regulation was reported in 60-80% of all AML, including also non-CBF-AML. Fischer et al identified that *miR-17,* *miR-18a*, *miR-20a* and *miR-93* all function as the CBF-AML fusion proteins in negative regulating their target RUNX1 and the *RUNX1-miR-221-KIT* axis [74]. *miR-17* up-regulation is associated with the M5 subtype AML, which is frequently characterized by KIT up-regulation. *miR-18a*, *miR-20a* and *miR-93* are frequently upregulated in distinct subtypes of non-CBF-AML [74]. Furthermore, this study found that increased KIT could delay myeloid differentiation. In the presence of factors that impair RUNX1 function/level this delay may act synergistically with deregulated of RUNX1-targets (e.g. *miR-223*) involved in differentiation of myeloid precursors. These data indicate that these miRNAs are linked to aberrant regulation of the network of *RUNX1-miRNAs* interactions underlying proliferation and myeloid differentiation in AML (Figure 3).

**Figure 4:**
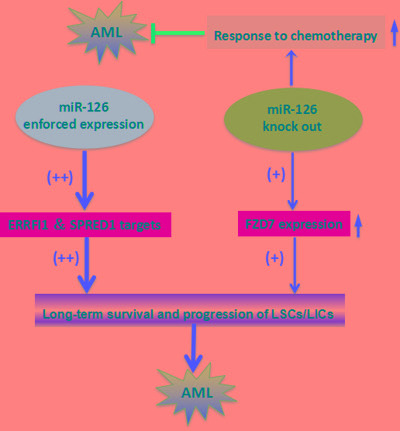
The two-faceted roles of miR126 in AML

Both gain- and loss-of-function *in vivo* studies of *miR-126* in mouse models demonstrated that either enforced expression or knockout of *miR-126* substantially promoted development of t(8;21) AML in mice [75]. *miR-126* overexpression in mice more drastically reduced long-term survival and increased progression of leukemia stem/initiating cells (LSCs/LICs) through the AML1-ETO9a pathway than the *miR-126* knockout. However, *miR-126* knockout leukemia cells were significantly more responsive to standard chemotherapy. *miR-126* overexpression leads to a gene expression profile similar to LSCs/LICs and/or primitive hematopoietic stem/progenitor cells by targeting ERRFI1 and SPRED1. *miR-126* knockout, on the other hand, yields a gene expression profile more similar to that seen in more differentiated hematopoietic progenitor cells presumably by inducing FZD7 expression. Together, these findings show that *miR-126* plays a dual role in leukemia, and uncovers a new layer of miRNA regulation in cancer (Figure 4).

*MLL-AF9* (alias *KMT2A-MLLT3*), a gene fusion product of chromosomal translocation t(9;11)(p22;q23), causes acute leukemia in mice [76]. It is the most common fusion gene found in infant cases of AML and correlates with monoblastic AML. 41 genes and 21 miRNAs with *MLL-AF9-*dependent expression were identified in endogenous *MLL-AF9* knockdown THP1 cells, derived from an AML patient [77, 78], including mediators of *MLL-AF9* leukemogenic effects. The miRNA-target genes were validated and gene ontology analysis implicated the up-regulated miRNAs’ targets in cell cycle progression and the downregulated miRNAs’ targets in cell cycle checkpoints, indicating that *MLL-AF9* via miRNAs promotes cell cycle progression and inhibits checkpoints. Stem cell maintenance and development and the stress response were among the processes predicted to be affected by miRNAs with *MLL-AF9* expression. Of these, 5 miRNAs (*miR-137*, *miR-214-3p*, *miR-301a-3p*, *miR-330-3p* and *miR-383-5p*) affect all four categories, linking them strongly to leukemia pathogenesis and highlighting their potential as therapeutic targets. *MLL-AF9* may cause leukemogenic effects by regulating both gene and miRNA expression. miRNA target genes were enriched in gene products related to the cell cycle, Wnt signaling, cell adhesion, myeloid differentiation, and the cellular stress response. These processes are likely directly regulated by *MLL-AF9* through downstream miRNA expression.

## MIRNAS AS POTENTIAL THERAPEUTIC TARGETS FOR AML

miRNAs hold great promise as potential cancer therapeutics but safe and specific delivery to the tumor sites remains the principle hurdle to their clinical implementation [79]. For the heterogeneous AMLs, it is difficult to optimize targeted therapies for each case. Instead, finding common downstream miRNA targets of the fusion gene related to AML might yield novel therapeutic options. Using miRNA-based therapeutics whole molecular signaling network, which act synergistically leading to a disease such as AML, can be targeted.

Immunotherapy has become an increasingly appealing therapeutic strategy for cancer patients. Blocking inhibitory immune checkpoint molecules (such as PD-1/PD-L1) enhance the immune response to tumors. An inverse correlation between PD-L1 and *miR-34a* expression has been observed in AML samples [80]. Over-expression of *miR-34a* in AML cell lines reduced PD-L1 mRNA and cell surface expression of PD-L1 protein. Further studies identified that the PD-L1 mRNA is a direct target of *miR-34a*, and PD-L1 specific T cell apoptosis could be reduced following *miR-34a* transfection, suggesting the potential of *miR-34a* mimics in cancer immunotherapy. Furthermore, a positive feedback between PD-L1 expression and AKT (also known as PKB, Protein Kinase B) activation was observed in the AML cell lines [80], and the activation of AKT-mTOR pathway augmented immune escape by driving expression of PD-L1 [81]. Increased AKT-mTOR pathway activity promotes survival of leukemia stem cells and early committed leukemic precursors and its inhibition provides a potential therapeutic approach. Studies demonstrated that *miR34a* over-expression profoundly decreased AKT phosphorylation level in multiple cancer types [82, 83], indicating the promising efficacy of *miR-34a* mimics in inhibiting both PD-L1 and AKT activity as an AML immunotherapy. Activation of PI3K/Akt/mTOR is activated in more than 60 % of AML patients and is associated with decreased overall survival [84]. In AML cell lines with AML1/ETO fusion protein, the *miR-193a* is down-regulated, and treatment of a leukemia mouse model with synthetic *miR-193a* results in significant tumor regression and reduction of AML1/ETO, CCND1, MDM2 as well as concomitant up-regulation of phosphatase and tensin homolog (PTEN) [85]. The *miR29* family members *miR29a*, -*29b* and *-29c* function as tumor suppressors in AML, regulating cell proliferation and apoptosis by the inhibiting AKT2 and CCND2, and show therapeutic potential for AML [22].

Inhibition of the ErbB pathway provides another potential new therapeutic target for leukemia [86, 87]. In AML NB4 cells ectopically expressing *miR-125a* ErbB pathway was significantly activated. In AML, ErbB receptors ErbB-1 and ErbB-3 were the main mediators of *miR-125a*, and the phosphorylation of the downstream effectors AKT and MAPK played key roles in driving proliferation and survival of the AML blasts. These findings indicate the potential of *miR-125a* as a new therapeutic target for miR-125a-low AML [10]. Furthermore, analysis of the upstream region of *miR-125a* and bisulfite sequencing revealed that *miR-125a* is suppressed by methylation in AML. The standard chemotherapy agent for AML, decitabine, also a de-methylating agent could significantly restore the expression of *miR-125a* through suppressing the global methylation in AML cell lines. It was known that elevated expression of ecotropic viral integration site 1 (Evi1) gene is associated with unfavorable prognosis in AML. In all, 5-10% of AML patients show Evi1 up-regulation. While Evi1-low patients showed >80% overall survival at 5 years, Evi1-high patients showed <60%. A recent study showed that *miR-133* binding to Evi1 increases drug sensitivity specifically in Evi1 expressing leukemic cells, suggesting that *miR-133* may be a promising therapeutic target for the Evi1 dys-regulated leukemia with poor prognosis [88]. Similarly, reduced expression of the *miR-9* target Hes1 has been to be an indicator of poor prognosis for AML [89]. Coincidentally, high expression of *miRNA-9* was identified in the leukemic progenitor cells (LPs) from CD34^+^ adult CN-AMLs [90]. Knockdown of *miR-9* in a mouse leukemia model suppressed AML cell proliferation, decreased leukemic cell counts in blood and bone marrow, reduced splenomegaly, and increased survival times, indicating that *miR-9* is a potential target for treatment of AML [90].

Su et al [37] demonstrated that *miR-181* inhibition is a potential new treatment strategy for AML. *miR-181* inhibits differentiation of AML cells into granulocytes and macrophages by down-regulating their direct targets *PRKCD*, *CTDSPL,* and *CAMKK1* and then affecting the PRKCD-P38-C/EBPα pathway and reducing pRB phosphorylation. Knockdown of *miR-181* in cultured bone marrow blasts from AML patients partially reversed blockage of myeloid differentiation. In AML CD34^+^ HSPC xenograft mice, inhibition of *miR-181* increased differentiation of myeloid progenitors, reduced engraftment and infiltration of leukemic HSPCs into bone marrow and spleen, and ameliorated symptoms of leukemia. These findings suggest that *miR-181* is a potential target for AML therapy. However, the temporal expression of *miR-181b* is also critical in determining the chemo-sensitivity in AML [91]. Human multidrug-resistant leukemia cells and relapsed/refractory AML patients have significantly lower levels of *miR-181b*. Leukemia cells overexpressing *miR-181b,* on the other hand, are more sensitive to cytotoxic chemotherapeutic agents and prone to drug-induced apoptosis. One of the underlying mechanisms is that *miR-181b* binds to the 3′-untranslated regions of *HMGB1* and *Mcl-1* and inhibits their expression. Direct suppression of HMGB1 sensitizes multidrug-resistant leukemia cells to chemotherapy and induces apoptotic cell death. These results demonstrate critical but complex roles of *miR-181* in AML, and more importantly, the temporal changes of miRNA expression and function during AML progression highlight a rigorous evaluation of miRNA-based therapy in AML. A comprehensive list of potential miRNA targets for AML therapy is summarized in Table 2.

**Table 2:** Potential miRNAs as treatment targets for AML

**miRNA**	**target genes or pathways**	**References**
miR-141	PI3K/Akt/mTOR	[113]
miR-125a	ErbB pathway	[10]
miR-125b	Mcl-1	[11, 12]
miR-22-3p, let-7e-5p	PLK1	[114]
miR-34a	PD-L1	[80]
miR-638	CDK2	[34]
miR-181a, b and c	PRKCD, CTDSPL and CAMKK1	[6, 36, 96, 97]
miR-191-5p, miR-142-3p	PPP2R2A	[19, 115]
miR-181b	MDR	[36]
miR-21, miR-196b	HOX	[98]
miR-29a/b/c	Dnmts	[19, 22]

## THE PROGNOSTIC VALUE OF MIRNAS IN AML

Both single miRNAs and panel of miRNAs have potential prognostic value complementing information gained from cytogenetics, gene mutations, and altered gene expression. Chen and his colleagues reported that reduced *miR-124-1* expression is commonly found in AML patients, most frequently in those with t(15;17). Patients with reduced *miR-124-1* expression tended to have slightly longer overall survival and relapse-free survival than those without reduced *miR-124-1*, suggesting that *miR-124-1* down-regulation predicts favorable survival in AML [92]. Liu et al reported that the down-regulation of circulating *miR-328* in AML patients correlates with poor clinical outcome, and may provide a diagnostic and prognostic biomarker [93]. Eisfeld et al reported *miR-3151* as an independent prognostic factor for outcome in older CN-AML patients [94]. In 179 CN-AML patients over 60 years of age high *miR-3151* expression was associated with shorter disease-free and overall survival; high expressing patients also had a lower CR rate compared with low expressers. In other work, patients with increased *miR-3151* had shorter overall and leukemia-free survival and higher cumulative incidence of relapse [95]. Moreover, multivariate analysis demonstrated that the combined observation of *miR-3151* and its host gene BAALC improved this prognostic stratification. It was reported that patients with low levels of both *miR-3151* and BAALC had better outcomes than patients with increased levels of either marker [95]. The down-regulation of *miR-181* was associated with leukemia invasiveness, and *miR-181* has been well studied to be a prognostic predictor of AML [6, 36, 96, 97]. It's shown that the expression of *miR-181a* and *miR-181b* was positively associated with good clinical outcome in molecular high-risk CN-AML and inversely associated with the risk of an event, such as failure to achieve complete remission, relapse, and even death.

In a study of intermediate-risk cytogenetic AML (IR-AML), increased *miR-196b* or *miR-644* were linked to shorter overall survival times, while reduced *miR-135a* and *miR-409-3p* were linked increased risk of relapse [98]. *miR-135a*, *miR-196b, miR-409-3p*, and *mir-644* were identified as prognostic markers for IR-AML, while *miR-122, miR-133b*, *miR-148a*, and *miR-409-3p* were found to be be valuable in prognosis of AML and linked to adverse outcomes for older CN-AML patients [8]. Lin et al reported that the high *let-7a-2-3p* and low *miR-188-5p* expression could be potentially used as favorably prognostic biomarkers independently in CN-AML patients [9]. Recently, a study reported that the high serum *miRNA-335* predicts poor outcomes and aggressive tumor progression in pediatric AML and may provide a prognostic indicator [99]. In another study, it was reported that *miR-212* is significantly associated with increased overall, event-free, and relapse-free survival. Moreover, it was found that the prognostic significance and the prevalence of high *miR-212* did not correlate with specific cytogenetic subtypes of AML, indicating that *miR-212* may improve the current prognostic risk stratification of mixed AML including normal karyotype AML and AML with cytogenetic and molecular abnormalities [100].

Recently, there are many other miRNAs were demonstrated to correlate with clinical outcomes of *de novo* adult AML patients and pediatric AML patients (Table 3). However, each study reported unique miRNAs and each miRNAs showed significant prognostic values in certain AML populations. These may further testify the complex and heterogeneous property of AMLs, and the lack of uniformity among all the studies could be explained by the heterogeneity in mutational profiles among AML patient cohorts. Taken together, miRNAs as prognostication of AML subtypes or subgroups warrants further study.

**Table 3:** miRNAs that implicate clinical prognosis in AML

**Prognosis status**	**Up-regulated miRNA**	**Down-regulated miRNA**	**References**
Good prognosis	miR-191, miR-199a, miR-30a, miR-30b, miR-30c, miR-181a, miR-181b, let-7a-2-3p	miR-124-1, miR-125, miR-126	[2, 6, 9-12, 33, 36, 53, 92, 96, 97, 105, 115]
Bad prognosis	miR-124, miR-128-1, miR-194, miR-219-5p, miR-220a, miR-320, miR-196b, miR-644, miR-3151, miR-146, miR-133b, miR-148a, miR-122, miR-409-3p, miR-126-5p/3p, miR-24, miR-331, miR-378	miR-150, miR-342, miR-135a, miR-409-3p, miR-96, miR-188-5p	[7-9, 53, 92, 94, 98, 105-107, 116-121]

## CONCLUDING REMARKS

miRNAs have emerged as major players in gene regulation underlying various aspects of AMLs. miRNAs and their regulatory signaling pathways must be placed alongside traditional protein-coding oncogenes and tumor suppressors if we aim to achieve a more thorough understanding of the complex mechanisms of malignant AML transformation. We are aware that a systems biological understanding of the miRNA regulatory networks is still superficial and limited. With in-depth studies, further clarifying the expression, function and regulatory mechanism of miRNAs will provide promising strategies for AML treatment.
